# Construct validity and reliability of the Test Your Memory Chinese version in older neurology outpatient attendees

**DOI:** 10.1186/s13033-018-0240-0

**Published:** 2018-10-26

**Authors:** Xuemei Li, Shengfa Zhang, Jinsui Zhang, Jingru Zhu, Huan He, Yurong Zhang, Weijun Zhang, Donghua Tian

**Affiliations:** 1 School of Social Development and Public Policy, Beijing Normal University, 19, Xinjiekou Wai Street, Beijing, 100875 China; 2 Clinics of Cadre, Department of Outpatient, General Hospital of the People’s Liberation Army (301 Hospital), Beijing, 100853 China; 3 School of Public Administration, Southwestern University of Finance and Economics, Chengdu, 611130 China

**Keywords:** Dementia, Alzheimer disease, Mild cognitive impairment, Test Your Memory, TYM

## Abstract

**Background:**

Early distinguishing the cognitive impairment from healthy population is crucial to delay the progression of mild cognitive impairment (MCI) and Alzheimer disease (AD). Test Your Memory (TYM) has been proved to be a valid and reliable screening instrument for AD and MCI. This study aimed to develop a culturally appropriate and functional Standard Mandarin Chinese translation of the TYM, and to evaluate its reliability and validity in detecting AD and MCI in Chinese.

**Methods:**

182 subjects with AD/MCI and 55 healthy controls were recruited to participate in this study, and everyone undergo the test of Standard Mandarin Chinese version of the TYM (TYM-CN), Mini-mental State Examination (MMSE), Montreal cognitive assessment (MoCA-BJ), and Clinical Dementia Rating (CDR) Scale. Concurrently, all the subjects with AD/MCI received the general physical and neurologic examinations, extensive laboratory tests, and brain computed tomography/magnetic resonance imaging (MRI). Of which, 90 subjects were asked to complete the re-test of TYM-CN at 3 weeks after the initial visit. Intra-class correlation coefficient (ICC) and Cronbach’s alpha was used to assess the test–retest reliability and the internal consistency. The validity, sensitivity and specificity were also analyzed. One-way analysis of variance, χ^2^ test, correlation analysis, and receiver operating characteristic curve (ROC) analysis were employed, as needed.

**Results:**

The total scores of TYM-CN was 43.89 ± 3.44, 40.88 ± 4.38, and 29.12 ± 7.44 (p < 0.01) for healthy controls group, MCI group, and AD group, respectively. The ICC for 11 items of TYM-CN ranged from 0.863 (copying) to 0.994 (anterograde), and that of the total scale was 0.993, suggesting an excellent reliability. Furthermore, the significant correlation was also found between TYM-CN and MMSE (r = 0.76), MoCA-BJ (r = 0.74), and CDR scores (r = 0.76), indicating a good validity. A TYM-CN scores ≤ 39.5 had 95% sensitivity and 95% specificity in differentiating AD from healthy controls, and that ≤ 43.5 had 75% sensitivity and 91% specificity in distinguishing MCI from healthy controls, respectively.

**Conclusion:**

The reliability and validity of the TYM-CN are statistically acceptable for the evaluation of cognitive impairment, which may contribute to neuropsychological tests for the diagnosis of AD and MCI from healthy controls in China.

## Background

Dementia and other cognitive problems are becoming an important public health concern with the increase of aging population worldwide. An estimated 44.4 million individuals in the world have dementia in 2013 and the number will be expected to reach an estimated 75.6 million by 2030 [1], and 115.4 million in 2050 [2]. In China, which has the largest population of people with dementia, the prevalence of dementia appears to have increased steadily between 1990 and 2010 with the aging intensification [1, 3, 4]. A recent article published in 2014 showed that the prevalence of dementia among individuals aged over 65 years was 5.14% in China [5]. Similarly, another recent review study, conducted by Prince and his colleagues, indicated that the age-standardized prevalence for individuals over 60 years varied in a narrow range of 5–7% in the most world regions, with a higher prevalence of 8.5% in Latin America and a distinctively lower prevalence of 2–4% in the four sub-Saharan African regions [2]. It was also noteworthy that 58% of all people with dementia lived in countries with low or middle incomes in 2010, and this proportion will continue to rise to 63% in 2030 and 71% in 2050 [2]. Dementia mainly includes several types: Alzheimer’s disease (AD), vascular dementia (VaD), dementia with Lewy bodies (DLB) and frontotemporal dementia (FTD), and AD is the commonest form of dementia, contributing to 50–75% of dementia cases [6]. In China, a notably higher prevalence of dementia and AD was found in rural areas than in urban ones [5]. Future projections should focus on the preventive interventions for lowering the incidence, the improvements in treatment and care for prolonging survival, and the disease-modifying interventions for preventing or slowing progression.

Mild cognitive impairment (MCI), which is associated with an increased risk of developing Alzheimer’s or other subtypes of dementia, affects many more people [2, 7], who always represents a transitional status between healthy aging and dementia. Its’ prevalence has been reported to be between 10 and 20% in people older than 65 years [8]. Therefore, the appraisal of a patient’s cognition is a crucial part of many medical consultations. Cognitive tests not only aid the diagnosis of dementia, but also contribute the medical and social management of patients [9]. The need for an early diagnosis of AD and other dementia has been widely recognized and supported by groups including the UK National Dementia Strategy [10] and the National Institute for Clinical Excellence (NICE) [11]. In reality, there was a long delay between symptom onset and diagnosis, varying from 8 to 32 months [12]. It’s well known that early diagnosis requires recognition of the first cognitive deficits seen in AD and MCI [13].

Once the effective treatments for Alzheimer’s disease are available, there will be an even greater need for a quick sensitive test that is suitable for use in primary care and by non-specialists. Therefore, a short standardized mental status examination, which meets the three critical requirements for widespread use by a non-specialist [9]: take minimal operator time to administer; test a reasonable range of cognitive functions; and be sensitive to mild Alzheimer’s disease, will be helpful for the assessment of cognitive function in subjects with memory impairment [14].

Currently, the Mini-Mental State Examination (MMSE) [15] and Montreal Cognitive Assessment (MoCA) [16] are the most commonly administered psychometric screening assessment of cognition in China and other countries. However, the former has serval disadvantages, such as the insensitivity to the earliest changes in highly educated individuals [17], the bias against visually impaired [18], and a lack of ability to measure frontal/executive function [14]. Although the latter had more sensitive at detection of mild dementia and a slightly better diagnostic accuracy than the former, it’s still has some bias against people with poor education [18], who have difficulty in completing a test. Therefore, these two tools don’t meet the three critical requirements above [9] for widespread use by a non-specialist. In the light of the above, Brown et al. [9] designed a brief test (Test Your Memory, TYM) for the detection of Alzheimer disease (AD) and amnestic mild cognitive impairment (aMCI) [13], which consists of a series of 10 self-administered tasks, and was reported to be a valid and reliable screening test for the detection of AD. Currently, TYM has been a powerful short cognitive test that examines verbal and visual recall and been a valuable addition to the assessment of patients with aMCI/AD [19]. Concurrently, TYM has been translated into different languages, such as Japanese [14], French [20], Spanish [21, 22], and Polish [23], and also presented a good psychometric properties and diagnostic capacity to identify case of dementia.

Primary care is a fundamental part of health care systems in both high and low income countries, and there was also ample evidence that primary care contributed to the improvement of health outcomes [24]. In rural China, primary care including township health center (THCs) and village clinics, have dramatically improved access to health care in the communities of rural China over the last few decades, and are still playing an important role in the rural health system. From 2009, Chinese government comprehensively implemented the national basic public health services projects. Village doctors and the medical workers in THCs are responsible for providing basic public health services, including the establishment of health records, chronic disease screening and management, severe mental disorders management, and health education, to rural residents. However, we could not ignore the fact that most of workers, especially village doctors, in primary cares of rural China did not received the professional training in medicine. Too complicated technology and screening tools were not easy to understand by them. Therefore, a simple, convenient, and powerful short cognitive assessment tool would better help them deliver public health services to residents, and further increase the accessibility and efficiency of public health services. Therefore, this study aimed to develop a culturally appropriate and functional Chinese version of the TYM (TYM-CN) and to evaluate its reliability and validity for measuring AD and mild cognitive impairment (MCI) in Chinese.

## Methods

### Instrument

The original version of TYM consists of 10 tasks on a double-sided sheet of card with spaces for the subjects to fill in response [9]. Specifically, the tasks include orientation (10 points), ability to copy a sentence (2 points), semantic knowledge (3 points), calculation (4 points), verbal fluency (4 points), similarities (4 points), naming (5 points), visuospatial abilities (2 tasks, total 7 points), and recall of the copied sentence (6 points) [9]. Additionally, the subjects’ ability to complete the test is also scored from 5 points for subjects requiring no help to 0 point for patients requiring major help as the 11th task [9, 20], this limitation of help is to ensure that the test is performed adequately in the process of filling the questionnaire. The total scores of TYM is 50 points.

### Translation and procedures

To obtain a Chinese version of TYM, multiple translation procedures were performed according to Beaton’s guideline, which was used for cross-cultural adaptation of health-related questionnaires [25]. The original version of TYM was firstly translated into a standard Mandarin Chinese version by two translators, one was a psychologist and another is a physician majoring in neurology. These two translators produced two primary TYM Chinese versions, independently. Subsequently, the third reconciled version was accomplished based on a comparison of the former two versions. On this basis, the fourth version was a translation of the Chinese version back into English and was produced by two additional translators, who had no the knowledge of the original questionnaire in advance. Eventually, any discrepancy of the fourth version from two translators was also resolved in the fifth version (the TYM-CN used in the present study) by an expert committee of School of Social Development and Public Policy at Beijing Normal University and the Department of Outpatient, General Hospital of the People’s Liberation Army (301 Hospital).

Cross-cultural adaptation was necessary in the process of translation of original TYM, therefore, the minor modifications were made when it was translated into Chinese. Specifically, the section about the ability to copying a sentence “good citizens always wear stout shoes” was adjusted to “Chinese working people always wear Jiefang shoes”, because the words “Jiefang shoes” is more familiar to Chinese people, especially, those over the age of 40. Furthermore, the words “Jiefang shoes” is much more vividly and specifically. The questions of semantic knowledge are “who is the prime minister?” and “In what year did the 1st World War start?” those are not common knowledge in China. However, Chinese people commonly know “the current national chairman” and “in what year People’s Republic of China was founded”. Therefore, these two questions were changed into “Who is the current national chairman of China?” and “The people’s Republic of China is founded in which year?” In the verbal fluency test, words beginning with the letter “S” were replaced by words beginning with the Chinese character ‘Hong’, which means red in Chinese. Chinese was familiar with this word, which was always on behalf of the happy, lucky, morale, happiness, etc. The last modification exists at the first question of visuospatial abilities test, the letter “W” was replaced by the Chinese character “Shang”. Chinese people, especially those in rural, are unfamiliar with the English letter, however, the vast majority of Chinese people know the word “Shang”. The other items were translated directly into Chinese.

In order to determine the readability and understandability of the preliminary TYM-CN, it was eventually distributed in a pilot study to 15 subjects, including 6 patients with mild AD, 4 patients with MCI, and 5 family members of patients with AD, who were asked about any unclear words, phrases, or concepts. The results of the pilot study showed that the TYM-CN could been easily understood by the subjects without significant complaints.

### Ethics approval and consent to participate

This study was approved by the Institutional Review Board (IRB) of School of Social Development and Public Policy (SSDPP) at Beijing Normal University (BNU). All subjects provided written informed consent.

### Subjects

Convenience sampling method, a type of nonrandom sampling, was used in this study. The main assumption associated with this sampling method is that the members of the target population are homogeneous [26]. Meanwhile, computing reliability and validity for questionnaires/scales need the minimum sample size. Based on the previous studies [27, 28], the minimum sample size in reliability and validity test should be at least 100, or the minimum ratios of sample size to the number of variables should be at least five.

A recent review study also found that about 90% of articles had a sample size greater than or equal to 100 for validating a scale [29]. In this study, we think that the minimum sample size of 100 subjects should be included based on the number of tasks of TYM-CN. Eventually, 182 subjects with AD or MCI were recruited from the patients attending the neurology outpatients from the General Hospital of the People’s Liberation Army (301 Hospital) between June 2014 and July 2015. Simultaneously, 55 healthy controls, who are family member of the patients above, were also included to this study.

Inclusion criteria included the following: (1) an ability to speak and read Chinese, (2) willing to cooperate with the investigators, and (3) agreeing to sign an informed consent form. While Exclusion criteria included: (1) the impaired verbal communication, visual impairment, (2) hearing-impaired, and mental retardation that could interfere with neuropsychological assessment, (3) underlying medical or psychiatric illness that could affect cognition, and absence of a reliable proxy.

The diagnosis of AD was based on the criteria which was published by the National Institute of Neurological and Communicative Disorders and Stroke and Alzheimer’s Disease and Related Disorders Association (NINCDS-ADRDA) [30] and the standard clinical diagnostic criteria for the diagnosis of dementia [31]. Additionally, the following criteria [32], were also used for MCI diagnosis of this study, including a CDR score (controls = 0, dementia ≥ 1, and MCI ≤ 0.5) [33–35], the absence of dementia, memory complaints by the patients or their family, normal global cognitive function, normal activities of daily living, and the objective impairment in memory as evident by scores more than 1.5 standard deviations (SD) below the age-appropriate mean. Beside above, all patients with AD/MCI received the detailed general physical, neurologic and psychiatric examinations, extensive laboratory tests, brain computed tomography (CT)/magnetic resonance imaging (MRI).

A neurologist at neurology department of 301 Hospital diagnosed dementia and MCI based on detailed neurological, neuropsychological, laboratory, and neuroimaging data for each subject. Eventually, one hundred and two subjects were diagnosed as having AD, eighty subjects were diagnosed as having MCI. Additionally, 55 healthy controls were recruited from family member of patients, based on the principle of voluntary participation.

### Neuropsychological tests

All the subjects underwent the Mini-mental State Examination (MMSE) [36–38], the Beijing version of Montreal cognitive assessment (MoCA-BJ) which was translated by Wei Wang and Hengge Xie from 301 Hospital [39–41], and the Chinese version of Test Your Memory (TYM-CN) by a three Ph.D. Candidates in psychometrics, under the guidance of physician in neurology. Clinical Dementia Rating (CDR) Scale was also used to assess the disease severity by a neurologist at neurology department. Concurrently, MMSE, MoCA-BJ, and TYM-CN tests were also administrated to the subjects in healthy controls. Of which, ninety subjects, including 70 patients with AD, 14 patients with MCI, and six healthy control, were asked to complete the re-test of TYM-CN by three nurses who didn’t know the subjects’ condition, 3 weeks after the initial visit, when they returned visit in the neurology outpatients. The demographic data of all subjects including gender, age, and educational level were also gathered in this study.

### Statistical analysis

The continuous data were described by using the means and standard deviation values, and the categorical data were presented by using frequencies and percentage. Differences in gender were analyzed by using the χ^2^ test. Between-group differences in age, years of education, and neuropsychological test scores were analyzed by using one-way analysis of variance (ANOVA) with a post hoc Bonferroni test. The test–retest reliability was quantified by using the intra-class correlation coefficient (ICC), Cronbach’s alpha was calculated to assess internal consistency, and the value above 0.70 was considered to be adequate [42]. Meanwhile, the validity of the scale was also assessed by calculating the correlations between scores of two tests, among TYM-CN, MMSE, MoCA-BJ, by using the Spearman rank correlation analysis. Furthermore, the correlations between the scores of TYM-CN and CDR was also evaluated. Eventually, the sensitivity and specificity of TYM-CN was also assessed by using the receiver operating characteristic curve (ROC) analysis. A statistical software SPSS v. 21 (IBM, Chicago, IL, USA) was used for analyses in this study.

## Results

### Demographic and clinical data

The total sample included 237 subjects including 158 males and 79 females. Table 1 summarized their demographic characteristics and clinical information based on the results of post hoc analysis. No significant differences (p > 0.05) were found among groups with respect to age [F_(2, 234)_ = 2.147, p = 0.119]. However, there were significantly difference in years of education [F_(2, 234)_ = 3.58, p = 0.029]. Additionally, three groups did differ significantly in global cognitive impairment of MMSE scores [F_(2, 234)_ = 108.39, p < 0.001] and MoCA scores [F_(2, 234)_ = 236.56, p < 0.001], and dementia severity [CDR: F_(2, 234)_ = 131.61, p < 0.001]. Furthermore, the subjects with dementia performed significantly worse than that with MCI and healthy controls, whereas the subjects with MCI performed significantly worse than healthy controls on the evaluation of global cognitive impairment and disease severity.

**Table 1:** Demographic data and cognitive screening tests by group (all subjects)

	AD, n = 102	MCI, n = 80	Control, n = 55	Dementia versus control	Dementia versus MCI	MCI versus control
Age	79.9 ± 6.49	78.01 ± 7.75	80 ± 6.07	ns	ns	ns
Gender						
Male	79 (77.5%)	58 (72.5%)	21 (38.2%)			
Female	23 (22.5%)	22 (27.5%)	34 (61.8%)			
Education level (years)	12.57 ± 4.19	12.73 ± 3.15	11.20 ± 4.19	**	ns	ns
MMSE	22.72 ± 3.95	27.30 ± 2.08	29.35 ± 1.16	**	**	**
MoCA-BJ	15.42 ± 4.40	22.79 ± 3.30	27.87 ± 1.74	**	**	**
CDR scale	1.12 ± 0.64	0.50 ± 0.00	0.00 ± 0.00	**	**	**

Results are expressed as mean ± SD

*MCI* mild cognitive impairment, *TYM-CN* Test Your Memory Chinese version

* p < 0.05, ** p < 0.01

### Comparisons of TYM-CN scores among three groups

The results of comparisons between the total TYM-CN scores and subscale scores for each group were shown in Table 2. The average total scores on the TYM-CN were significantly lower in AD and MCI groups than in the healthy controls group, and also significantly lower in AD group than in MCI group. The group of subjects with AD showed significantly lower scores on all the subscales including orientation, the ability to copy a sentence, semantic knowledge, calculation, verbal fluency, similarities, naming, visuospatial abilities, anterograde memory, and executive function, than MCI and healthy controls group. Additionally, the group of subjects with MCI has also significantly lower scores in most of subscales, except for orientation, copying, and naming.

**Table 2:** Comparison of performance on TYM-CN (total and subscale scores) between three groups

	AD, n = 102	MCI, n = 80	Control, n = 55	Dementia versus control	Dementia versus MCI	MCI versus control
Orientation (10)	7.13 ± 2.40	8.41 ± 2.15	8.55 ± 2.50	**	**	ns
Copying (2)	1.86 ± 0.40	1.98 ± 0.16	2.00 ± 0.00	*	*	ns
Semantic knowledge (3)	1.62 ± 0.86	2.71 ± 0.46	3.00 ± 0.00	**	**	**
Calculation (4)	3.02 ± 1.10	3.65 ± 0.73	3.98 ± 0.14	**	**	*
Verbal fluency (4)	2.19 ± 1.14	2.95 ± 0.98	3.38 ± 0.59	**	**	*
Similarities (4)	2.01 ± 1.38	3.63 ± 0.66	3.98 ± 0.14	**	**	**
Naming (5)	3.20 ± 1.84	3.93 ± 1.67	3.36 ± 1.28	**	*	ns
Visuospatial 1 (3)	0.69 ± 1.26	1.94 ± 1.44	2.89 ± 0.57	**	**	**
Visuospatial 2 (4)	2.25 ± 1.61	3.61 ± 0.85	3.93 ± 0.33	**	**	*
Anterograde (6)	1.12 ± 1.75	4.03 ± 1.64	4.65 ± 1.31	**	**	*
Executive (help) (5)	2.94 ± 1.33	3.79 ± 1.27	4.16 ± 0.79	**	**	*
TYM-CN	29.12 ± 7.44	40.88 ± 4.38	43.89 ± 3.44	**	**	**

Results are expressed as mean ± SD

*MCI* mild cognitive impairment, *TYM-CN* Test Your Memory Chinese version

* p < 0.05, ** p < 0.01

### Effect of age, education, and gender on TYM-CN scores

In this study, age showed a weak correlation with the TYM-CN score within the healthy controls group (Kendall’s tau = − 0.25, p = 0.016), but not within the AD groups (Kendall’s tau = − 0.05, p = 0.58) and MCI group (Kendall’s tau = − 0.02, p = 0.80). As for subscales, there was only a weak evidence that the scores of calculation and naming of the TYM-CN varied with age. The years of education showed a weak positive correlation with the TYM-CN score within the MCI group (Kendall’s tau = 0.23, p = 0.008), but not within the AD groups (Kendall’s tau = 0.05, p = 0.55) and healthy controls group (Kendall’s tau = 0.14, p = 0.20). Concurrently, there was also a weak correlation in between the years of education and the scores of semantic knowledge (Kendall’s tau = 0.15, p = 0.006), naming (Kendall’s tau = 0.24, p < 0.001), and Visuospatial 1 (Kendall’s tau = 0.20, p = 0.001). Additionally, a weak positive correlation was found between male and the total scores of TYM-CN in AD group (Kendall’s tau = 0.19, p = 0.02) and MCI group (Kendall’s tau = 0.19, p = 0.049). Furthermore, a weak correlation in between gender and the scores of semantic knowledge (Kendall’s tau = − 0.17, p = 0.006), verbal fluency (Kendall’s tau = − 0.12, p = 0.05), similarities (Kendall’s tau = − 0.21, p = 0.001), naming (Kendall’s tau = 0.17, p = 0.005), visuospatial 1 (Kendall’s tau = − 0.27, p < 0.001), and anterograde (Kendall’s tau = − 0.13, p = 0.027). However, these results may be attributed to the sex ratio imbalance, because the male female ratio was 2:1 in this study.

### Reliability analysis of TYM-CN

The results of reliability analysis showed that the ICC for 11 subscales was highly correlated between the test–retest among ninety subjects, with a range from 0.863 (copying) to 0.994 (anterograde) (Table 3), indicating an excellent reliability between the test–retest. Furthermore, the ICC for the total TYM-CN also presented excellent reliability with a value of 0.993. Cronbach’s alpha coefficient was 0.994 for total scores and 0.843 above for each subscale, also suggesting an excellent internal consistency for the total scale and subscales of the TYM-CN at the primary and secondary visits, respectively. Additionally, for all subjects, Cronbach’s alpha coefficient was 0.739, suggesting a good internal consistency for the 11 items of TYM-CN.

**Table 3:** ICC between the test–retest of the TYM-CN

Subscale	T1 (n = 90)	T2 (n = 90)	ICC	95% CI	p-value
Orientation	7.27 ± 2.05	7.34 ± 2.15	0.965	0.946–0.977	< 0.001
Copying	1.60 ± 0.51	1.53 ± 0.52	0.863	0.791–0.910	< 0.001
Semantic knowledge	2.03 ± 1.13	2.00 ± 1.13	0.980	0.970–0.987	< 0.001
Calculation	2.96 ± 1.35	2.90 ± 1.31	0.979	0.969–0.986	< 0.001
Verbal fluency	2.56 ± 1.48	2.63 ± 1.34	0.973	0.959–0.982	< 0.001
Similarities	2.31 ± 1.69	2.43 ± 1.60	0.984	0.975–0.989	< 0.001
Naming	3.44 ± 1.81	3.57 ± 1.66	0.985	0.978–0.990	< 0.001
Visuospatial 1	1.80 ± 0.86	1.70 ± 0.94	0.893	0.837–0.930	< 0.001
Visuospatial 2	2.13 ± 1.51	2.27 ± 1.47	0.968	0.952–0.979	< 0.001
Anterograde	2.07 ± 2.60	2.13 ± 2.57	0.994	0.991–0.996	< 0.001
Executive (help)	2.78 ± 1.56	2.95 ± 1.49	0.961	0.941–0.974	< 0.001
TYM-CN total	29.91 ± 9.70	30.69 ± 10.16	0.993	0.989–0.995	< 0.001

*T1* test, *T2* retest, *SD* standard deviation, *ICC* intraclass correlation coefficient, *CI* confidence interval

### Validity analysis of TYM-CN

The total TYM-CN scores was significantly correlated with scores of MMSE (r = 0.76, p < 0.0001) and MoCA-BJ (r = 0.74, p < 0.0001). Furthermore, the total TYM-CN scores was also significantly correlated with CDR scores (r = 0.76, p < 0.0001), which assessed the disease severity.

### Sensitivity and specificity of TYM-CN

**Fig. 1:**
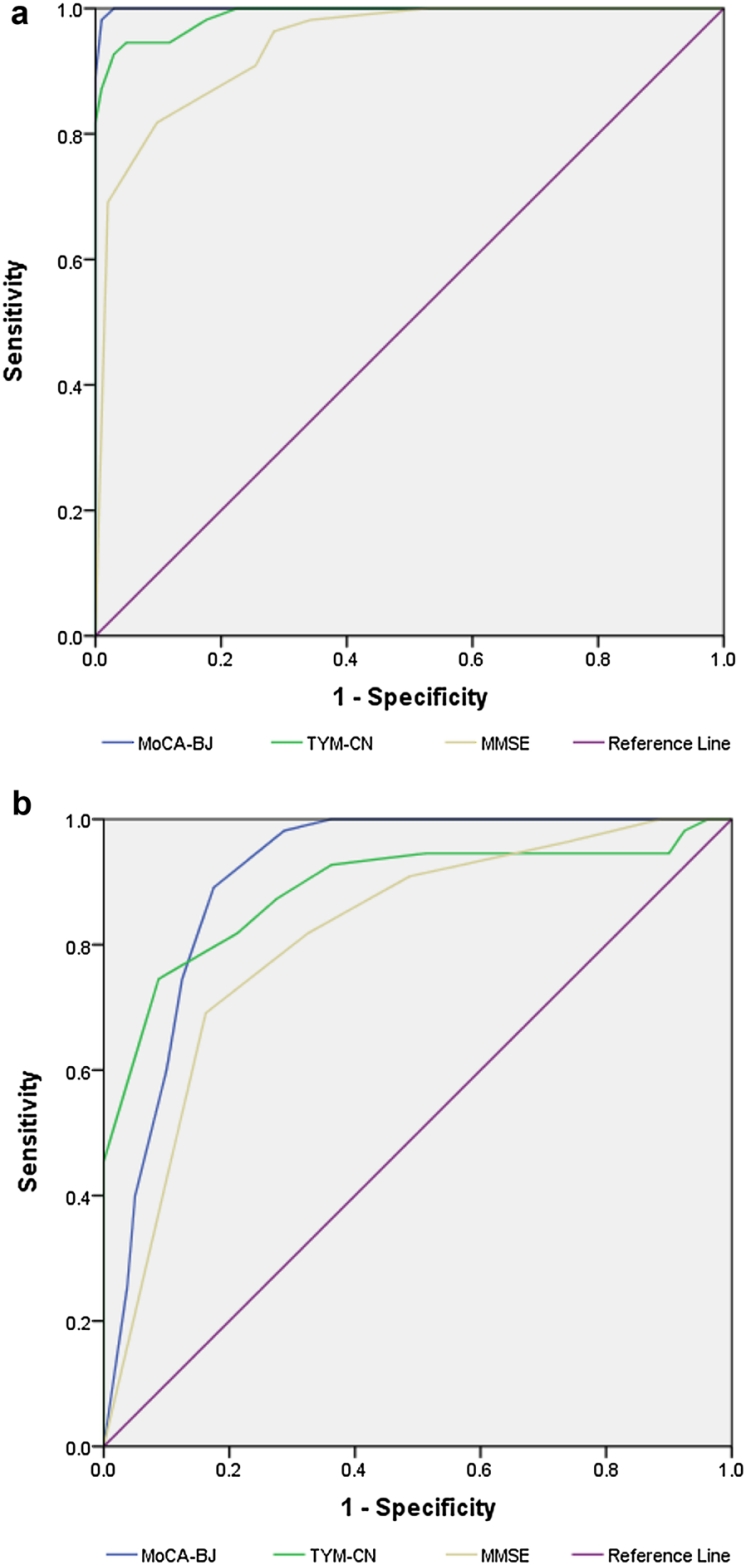
Receiver operating characteristic (ROC) curves for the TYM-CN, MMSE, and MoCA-BJ screening tests in differentiating AD (**a**) and MCI (**b**) from health controls. TYM-CN, TYM Chinese version; MMSE, the Mini-mental State Examination; MoCA-BJ, Beijing version of Montreal cognitive assessment

The diagnostic utility of TYM-CN with that of both MMSE and MoCA-BJ, which examine several cognitive domains, were also compared in this study. We found there was a significant difference in the Area Under the Curve (AUC) in between TYM-CN and MMSE, and in between TYM-CN and MoCA-BJ, and the sensitivity of TYM-CN fell in between MoCA-BJ and MMSE. Figure 1 showed the ROC curves which discriminated between AD group and healthy control group (Fig. 1a) and between MCI group and healthy controls group (Fig. 1b), respectively. ROC analysis demonstrated that the AUC was 0.989 (95% CI 0.977–1.00) for the TYM-CN, 0.999 (95% CI 0.997–1.000) for MoCA-BJ, and 0.941 (95% CI 0.907–0.976) for MMSE in differentiating AD from healthy controls group (Fig. 1a). Table 4 illustrated the sensitivity and specificity for the diagnosis of AD with different cut-offs of the TYM-CN. TYM-CN achieved the best differentiation between AD group and healthy control group for a cut-off value of ≤ 39.5, with a sensitivity and specificity of 95% and 95%, respectively. The sensitivity and specificity of the MMSE were 81.8% and 90%, respectively, with the established cut off ≤ 24. It should be noted that MoCA-BJ also presented the excellent diagnostic utility for AD, the sensitivity and specificity of the mini-mental state examination were 98.2% and 90%, with the established cut off ≤ 23.5. Additionally, Fig. 1b showed the results of ROC analysis for MCI group and healthy control group, the AUC was 0.887 (95% CI 0.824–0.951) for the TYM-CN, 0.909 (95% CI 0.859–0.959) for MoCA-BJ, and 0.813 (95% CI 0.739–0.887) for MMSE in differentiating MCI group from healthy control group. Table 5 illustrated the sensitivity and specificity for the diagnosis of MCI with different cut-offs of the TYM-CN. The TYM-CN differentiated MCI group from healthy controls group for a cut-off value of ≤ 43.5, with a sensitivity and specificity of 75% and 91%, respectively. Similarly, MoCA-BJ also presented the excellent diagnostic utility for MCI, the sensitivity and specificity were 75% and 87% with the established cut off ≤ 25.5. However, MMSE didn’t present a good performance in distinguishing MCI from health controls, the sensitivity and specificity were 81.8% and 67.5%, respectively, with the established cut off ≤ 24.5. These findings suggested that the TYM-CN may be a powerful diagnostic instrument for AD and MCI in Chinese.

**Table 4:** Sensitivity and specificity of optimal cut-off scores for diagnosis of AD

TYM-CN cut-off	Sensitivity	Specificity	PPV	NPV
≤ 34.5	98	82	99	75
≤ 35.5	95	88	97	81
≤ 37	95	92	97	87
≤ 38.5	95	94	97	90
≤ *39.5*	*95*	*95*	*97*	*90*
≤ 40.5	93	97	98	94
≤ 41.5	87	90	94	98

Numbers represent percentages

**Table 5:** Sensitivity and specificity of optimal cut-off scores for diagnosis of MCI

TYM-CN cut-off	Sensitivity	Specificity	PPV	NPV
≤ 41.5	87	73	92	69
≤ 42.5	82	79	86	73
≤ *43.5*	*75*	*91*	*84*	*85*

Numbers represent percentages

## Discussion

To the best of our knowledge, we most likely provided the first report of the validity and reliability of the TYM-CN for the evaluation of cognitive impairment in subjects with AD or MCI in a certain Chinese populations. The reliability and validity of the TYM-CN were confirmed by using an internal consistency analysis and correlation analysis, respectively. Concurrently, the diagnostic utility was also evaluated by using ROC analysis. The ICC, calculated by the test–retest method, indicated the excellent reliability with values of 0.863–0.994 (Table 3), was consistent with the results of the original study conducted by Brown et al. [9].

Cultural adaptation will be necessary in the process of cross-cultural translation of scales in some researches. In this study, TYM-CN was modified with the minor adjustments to the copying, the semantic knowledge question, fluency test, and visuospatial abilities, taking into account cultural differences. Specifically, the sentence of “good citizens always wear stout shoes” about the ability to copying a sentence was adjusted to “Chinese working people always wear Jiefang shoes”, considering that “Jiefang shoes” is more familiar to Chinese people. Similarly, most of Chinese people could not answer the questions of semantic knowledge “who is the prime minister?” and “In what year did the 1st World War start?” because those are not common knowledge in China. However, almost all Chinese people know “the current national chairman” and “in what year People’s Republic of China was founded”. Therefore, the original two questions were changed to the latter two questions. Additionally, in the verbal fluency test, words beginning with the letter “S” were replaced by the Chinese character words ‘Hong’, which means red color, and sometimes symbolizes the happy, lucky, morale, and happiness in Chinese. The first question of visuospatial abilities test was also modified, namely, the letter “W” was replaced by the Chinese character words “Shang”. Chinese old people, especially in rural, are unfamiliar with the English letter, however, the vast majority of Chinese people know the word “Shang”.

Being consistent with the English original, we found good correlations between the TYM-CN and other neuropsychological tests. Strong and statistically significant correlation, which was found between the TYM-CN and the other measures of global cognitive impairment (MMSE and MoCA-BJ) and dementia severity (CDR), supported the content validity of TYM-CN. These results were consistent with those reported previously by the scientists in Japan [14], Span [22], France [20], Poland [23], and South Africa [43] by using this scale in their respective native languages, which have also reported acceptable correlations between the TYM and other cognitive measures. These findings also suggested that the original English version of the TYM could be applied cross-culturally for the evaluation of cognitive impairment.

The important correlation between the TYM-CN and other global cognitive impairment measures, such as MMSE and MoCA-BJ used widely in China, suggested that the TYM should be sensitive to executive disorders. Furthermore, we also thought that an administration time of approximately 10 min should be acceptable for a screening test in outpatients or clinics in primary health centers, because the MMSE also takes an average of 8 to 10 min to administer. However, MMSE was insensitive to the earliest changes of subjects with high education level [17]. Furthermore, the sensitivity of TYM-CN was better than MMSE in this study. As Brown put forward earlier [9], a screening instrument, meeting the three critical requirements, including the minimal operator time, a reasonable range of cognitive functions, and the sensitivity to mild Alzheimer’s disease, for widespread use by a non-specialist would contribute to early diagnosis of certain dementias. Therefore, such tools should be included in the cognitive tools and should be encouraged to use widely in practices.

In this study, the average total TYM-CN scores were similar to those of the English original TYM, which were 43.9 and 46.6/44.1 [9, 19] in healthy controls group, 40.9 and 36.3 [19] in subjects with MCI, and 29.12 and 29.5/33.2 [9, 19] in subjects with AD, respectively. Besides the English original TYM, the results were in good agreement with those reported by other scientists [14, 21, 22, 44]. We also noted that there were slight differences in each items scores between the Chinese version and the English original in patients with healthy control and AD, however, these two tests indicated that subjects with AD had particularly impaired anterograde memory compared with healthy controls. Furthermore, the significant discrepancy were also found in the scores of each subscale in between AD and healthy controls, and in between AD and MCI. This also indicated that cognitive impairments deteriorated gradually from healthy controls to MCI and to AD. As for the results of comparison between healthy controls and MCI, most of items presented the significant difference except for the subscale of orientation, copying, and naming, this was most likely due to the differences in age and severity of cognitive dysfunction.

The diagnostic utility of the TYM-CN to differentiate the cases of AD and MCI from healthy controls was supported by the high AUC and its acceptable sensitivity and specificity. In this study, TYM-CN differentiate significantly better between the AD group and the healthy controls than the MMSE, with the optimal cut-off scores (39.5), both the sensitivity and specificity for diagnosis of AD were 95%. Although, the cut-off score were lower than that of English original TYM with the cut-off score (42/43), the sensitivity of 93%, and specificity of 86% [9], our results also supported that TYM-CN performed better than MMSE in distinguishing AD from healthy controls. Meanwhile, TYM-CN also distinguished significantly MCI from healthy controls with the optimal cut-off scores (43.5), the sensitivity and specificity for diagnosis of MCI were 75% and 91%, respectively.

As we expected that MMSE also discriminated AD from healthy controls with the sensitivity of 81.8% and specificity of 90%, at the established cut off of ≤ 24. However, it did not exhibit the good ability in distinguishing MCI from the healthy controls. An early study, conducted by Trzepacz et al., also indicated that MoCA and MMSE were more similar for dementia cases, however MMSE didn’t distinguish MCI cases [45]. This further illustrated that MMSE was insensitive to the earliest changes in highly educated individuals [17]. Additionally, the mean education level of subjects in this study was 12.28 years, which may also influence the performance of MMSE. It must be mentioned that the sensitivity of MoCA in the detection of mild dementia and early cognitive impairment has been well known [46, 47]. Indeed, MoCA-BJ also presented a good ability in discriminating AD and MCI from health controls, and the order of the AUC was the following: AUC_MoCA-BJ _> AUC_TYM-CN _> AUC_MMSE_ for differentiating AD and MCI from health controls in this study. A previous study also showed that the MoCA was a better cognitive tool than the widely used MMSE for the screening and monitoring of MCI and AD in clinical settings [48]. However, the level of difficulty of the items of MoCA-BJ was more than that of TYM-CN, therefore, it also took a long time in completing the evaluation in this study. Previous studies also reported that MoCA has some bias against people with poor educations, who have difficulty in completing a test [18, 49]. Actually, most of individuals over 60 years old in rural China did not got a good educations, this may limit the use of MoCA in these population. Concurrently, this disadvantage may also limit its application by the non-specialists at the grass-root health care, especially, in rural area. In comparison, the advantage of TYM became apparent, it was a self-administered test with a brief but rigorous scoring system, excellent inter-rater agreement for scoring, short time to completion, and can also be used easily by non-specialists, besides the advantage of cognitive tests. From this perspective, TYM-CN test not only can be used by the professionals in hospital, but also can be used by non-specialists at the village clinics in rural China.

## Limitations of this study

Several limitations existed in the present study. First, a small convenience sample from the outpatient of one hospital was employed, furthermore, the subjects were older with mean age of 79 years old, although, there’re no difference in age among three groups. Therefore, it could preclude a generalization of the results obtained to an unselected population. Second, sex ratio imbalance (male female ratio of 2:1) was another limitation of this study, there was a weak correlation in between gender and the scores of subscale in TYM-CN, which may be originated from the gender differences. Third, the number of healthy controls was small, furthermore, the subjects in healthy controls group not only were younger than that in AD group, but also has less educational level than that in AD group, these limitations may influence the confirmation of cut-off value. Further studies of large sample size are needed to determine the extent of susceptibility to cultural (such as different ethnic and regions), educational, and age bias in our research team in the future.

Of course, there were several strengths which should be highlighted. First, all the patients with AD and MCI were diagnosed by a neurologist at neurology department of 301 Hospital, which is one of the highest level of general hospital in China, according to the detailed clinical examination data of each subject, based on criteria of NINCDS-ADRDA). Second, CDR scores were also employed by the nurses at neurology department of 301 Hospital under the guidance of the neurologist. Third, to better perform the reliability analysis, 90 subjects were asked to complete the re-test of TYM-CN by other three nurses who didn’t know patients’ condition at 3 weeks after the initial visit. These strengths undoubtedly enhanced the credibility of the results.

## Conclusion

To the best of our knowledge, this may be the first study on the validity and reliability of the TYM-CN in China. We found that the TYM-CN was a valid and reliable instrument, which could contribute to the diagnosis of AD and MCI from the healthy controls. Furthermore, it promises to be a screening test by a non-specialist in primary cares in China in the future.

## References

[1] Junfang X, Wang J, Wimo A, Fratiglioni L, Fratiglioni L, Qiu C (2017). The economic burden of dementia in China, 1990–2030: implications for health policy. Bull World Health Organ.

[2] Prince M, Bryce R, Albanese E, Wimo A, Ribeiro W, Ferri CP (2013). The global prevalence of dementia: a systematic review and metaanalysis. Alzheimers Dement.

[3] Zhang Y, Xu Y, Nie H, Lei T, Wu Y, Zhang L, Zhang M (2012). Prevalence of dementia and major dementia subtypes in the Chinese populations: a meta-analysis of dementia prevalence surveys, 1980-2010. J Clin Neurosci.

[4] Chan KY, Wang W, Wu JJ, Liu L, Theodoratou E, Car J, Middleton L, Russ TC, Deary IJ, Campbell H (2013). Epidemiology of Alzheimer’s disease and other forms of dementia in China, 1990–2010: a systematic review and analysis. Lancet.

[5] Jia J, Wang F, Wei C, Zhou A, Jia X, Li F, Tang M, Chu L, Zhou Y, Zhou C (2014). The prevalence of dementia in urban and rural areas of China. Alzheimers Dement.

[6] Alzheimer’s Disease International (ADI). World Alzheimer Report 2009: the Global Prevalence of Dementia. London: Alzheimer’s Disease International; 2009.

[7] Yanhong O, Chandra M, Venkatesh D (2013). Mild cognitive impairment in adult: a neuropsychological review. Ann Indian Acad Neurol.

[8] Petersen RC, Roberts RO, Knopman DS, Geda YE, Cha RH, Pankratz VS, Boeve BF, Tangalos EG, Ivnik RJ, Rocca WA (2010). Prevalence of mild cognitive impairment is higher in men. The Mayo Clinic Study of Aging. Neurology.

[9] Brown J, Pengas G, Dawson K, Brown LA, Clatworthy P (2009). Self administered cognitive screening test (TYM) for detection of Alzheimer’s disease: cross sectional study. BMJ.

[10] Department of Health (2009). Living well with dementia: a national dementia strategy.

[11] (NICE) NIfHaCE (2006). Dementia: supporting people with dementia and their carers in health and social care.

[12] Bond J, Stave C, Sganga A, O’Connell B, Stanley RL (2005). Inequalities in dementia care across Europe: key findings of the facing dementia survey. Int J Clin Pract Suppl.

[13] Brown JM, Wiggins J, Dong H, Harvey R, Richardson F, Hunter K, Dawson K, Parker RA (2014). The hard Test Your Memory. Evaluation of a short cognitive test to detect mild Alzheimer’s disease and amnestic mild cognitive impairment. Int J Geriatr Psychiatry.

[14] Hanyu H, Maezono M, Sakurai H, Kume K, Kanetaka H, Iwamoto T (2011). Japanese version of the Test Your Memory as a screening test in a Japanese memory clinic. Psychiatry Res.

[15] Black LJ (1975). Complimentary, sequential antifertility effects of chlormadinone and norethindrone in the rabbit: implications in progestin only fertility control. Contraception.

[16] Nasreddine ZS, Phillips NA, Bedirian V, Charbonneau S, Whitehead V, Collin I, Cummings JL, Chertkow H (2005). The Montreal Cognitive Assessment, MoCA: a brief screening tool for mild cognitive impairment. J Am Geriatr Soc.

[17] O’Bryant SE, Humphreys JD, Smith GE, Ivnik RJ, Graff-Radford NR, Petersen RC, Lucas JA (2008). Detecting dementia with the mini-mental state examination in highly educated individuals. Arch Neurol.

[18] Oxford Medical Education (2017). Cognitive function tests in dementia.

[19] Brown JM, Lansdall CJ, Wiggins J, Dawson KE, Hunter K, Rowe JB, Parker RA (2017). The Test Your Memory for mild cognitive impairment (TYM-MCI). J Neurol Neurosurg Psychiatry.

[20] Postel-Vinay N, Hanon O, Clerson P, Brown JM, Menard J, Paillaud E, Alonso E, Pasquier F, Pariel S, Belliard S (2014). Validation of the Test Your Memory (F-TYM Test) in a French memory clinic population. Clin Neuropsychol.

[21] Ferrero-Arias J, Turrion-Rojo MA (2016). Validation of a Spanish version of the Test Your Memory. Neurologia.

[22] Munoz-Neira C, Henriquez Chaparro F, Delgado C, Brown J, Slachevsky A (2014). Test Your Memory-Spanish version (TYM-S): a validation study of a self-administered cognitive screening test. Int J Geriatr Psychiatry.

[23] Szczesniak D, Wojtynska R, Rymaszewska J (2013). Test Your Memory (TYM) as a screening instrument in clinical practice—the Polish validation study. Aging Ment Health.

[24] Starfield B (1994). Is primary care essential?. Lancet.

[25] Beaton DE, Bombardier C, Guillemin F, Ferraz MB (2000). Guidelines for the process of cross-cultural adaptation of self-report measures. Spine (Phila Pa 1976).

[26] Suen LJ, Huang HM, Lee HH (2014). A comparison of convenience sampling and purposive sampling. Hu Li Za Zhi.

[27] MacCallum RC, Widaman KF, Zhang S, Hong S (1999). Sample size in factor analysis. Psychol Methods.

[28] Gorsuch RL (1983). Factor analysis.

[29] Anthoine E, Moret L, Regnault A, Sebille V, Hardouin JB (2014). Sample size used to validate a scale: a review of publications on newly-developed patient reported outcomes measures. Health Q Life Outcomes.

[30] McKhann G, Drachman D, Folstein M, Katzman R, Price D, Stadlan EM (1984). Clinical diagnosis of Alzheimer’s disease: report of the NINCDS-ADRDA Work Group under the auspices of Department of Health and Human Services Task Force on Alzheimer’s Disease. Neurology.

[31] American Psychiatric Association (2000). Diagnostic and statistical manual of mental disorders (4th Edition) (DSM-IV-TR).

[32] Petersen RC, Doody R, Kurz A, Mohs RC, Morris JC, Rabins PV, Ritchie K, Rossor M, Thal L, Winblad B (2001). Current concepts in mild cognitive impairment. Arch Neurol.

[33] Hughes CP, Berg L, Danziger WL, Coben LA, Martin RL (1982). A new clinical scale for the staging of dementia. Br J Psychiatry.

[34] Gao Z, Wang W, Shang Y, Bai X, Wu W (2011). Exploration of the Chinese version montreal cognitive assessment in the diagnosis of mild cognitive impairment. Chin J Health Care Med.

[35] Morris JC (1993). The Clinical Dementia Rating (CDR): current version and scoring rules. Neurology.

[36] Folstein MF, Folstein SE, McHugh PR (1975). “Mini-mental state”. A practical method for grading the cognitive state of patients for the clinician. J Psychiatr Res.

[37] Gao M, Yang M, Kuang W, Qiu P (2015). Factors and validity analysis of Mini-Mental State Examination in Chinese elderly people. J Peking Univ (Health Sciences).

[38] Peng D, Xu X, Liu J, Jiao Y, Zhang H, Yin J, Meng X, Xie Y, Feng K (2005). Discussion on application of MMSE for senile dementia patients. Chin J Neuroimmunol Neurol.

[39] Sun H, Xie Y, Zhang X, Xie H, Wu W. Items in montreal cognitive assessment. Chin J Geriatr Heart Brain Vessel Dis. 2014:387–90 **(in Chinese)**.

[40] Xie H (2010). Cognitive impairment and neuropsychological assessment in Alzheimer’s disease. Chin J Pract Intern Med.

[41] Huang F, Wang Y, Li J, Wang L, Jiang Y, Liao S (2017). Diagnostic value of montreal cognitive assessment for mild cognitive impairment in Chinese middle-aged adults: a meta-analysis. Chin J Evid Based Med..

[42] Nunnally JC, Bernstein IH (1994). Psychometric theory.

[43] van Schalkwyk G, Botha H, Seedat S (2012). Comparison of 2 dementia screeners, the Test Your Memory Test and the Mini-Mental State Examination, in a primary care setting. J Geriatr Psychiatry Neurol.

[44] Papachristou E, Ramsay SE, Papacosta O, Lennon LT, Iliffe S, Whincup PH, Goya Wannamethee S (2016). The Test Your Memory cognitive screening tool: sociodemographic and cardiometabolic risk correlates in a population-based study of older British men. Int J Geriatr Psychiatry.

[45] Trzepacz PT, Hochstetler H, Wang S, Walker B, Saykin AJ, Alzheimer’s Disease Neuroimaging I (2015). Relationship between the Montreal Cognitive Assessment and Mini-mental State Examination for assessment of mild cognitive impairment in older adults. BMC Geriatr.

[46] O’Caoimh R, Timmons S, Molloy DW (2016). Screening for mild cognitive impairment: comparison of “MCI Specific” Screening Instruments. J Alzheimers Dis.

[47] Hoops S, Nazem S, Siderowf AD, Duda JE, Xie SX, Stern MB, Weintraub D (2009). Validity of the MoCA and MMSE in the detection of MCI and dementia in Parkinson disease. Neurology.

[48] Freitas S, Simoes MR, Alves L, Santana I (2013). Montreal cognitive assessment: validation study for mild cognitive impairment and Alzheimer disease. Alzheimer Dis Assoc Disord.

[49] Gomez F, Zunzunegui M, Lord C, Alvarado B, Garcia A (2013). Applicability of the MoCA-S test in populations with little education in Colombia. Int J Geriatr Psychiatry.

